# PD222 Balancing Patient Preferences With Feasible Healthcare Delivery: Using Discrete Choice Experiments Alongside Knowledge Exchange To Inform Care Pathways

**DOI:** 10.1017/S0266462324004410

**Published:** 2025-01-07

**Authors:** Emily Holmes, Beth Morris, Pete Dixon, Amy Mathieson, Leone Ridsdale, Myfanwy Morgan, Alison McKinlay, John Dickson, Steve Goodacre, Mike Jackson, Dyfrig Hughes, Tony Marson, Adam Noble

## Abstract

**Introduction:**

Emergency department (ED) visits for epilepsy are common, costly, and often clinically unnecessary. Configuration of care pathways (CPs) that could divert people away from ED offer an alternative. The aim was to measure patient and carer preferences for alternative CPs and to explore the feasibility of implementing the preferred CPs in the National Health Service (NHS) England with a wider group of stakeholders.

**Methods:**

Formative work (provider survey, service-user interviews, knowledge exchange, and think-aloud piloting) informed a discrete choice experiment (DCE) with six attributes: access to care plan, conveyance, time, epilepsy specialist today, general practitioner (GP) notification, and epilepsy specialist follow-up. This was hosted online with random assignment to two of three scenarios (home, public, or atypical). Logistic regression generated preference weights that were used to calculate the utility of CPs. The highest ranked CPs plus a status quo were discussed at three online knowledge exchange workshops. The nominal group technique was used to ascertain stakeholder views on preference evidence and to seek group consensus on optimal feasible alternatives.

**Results:**

A sample of 427 people with epilepsy and 167 friends or family completed the survey. People with epilepsy preferred paramedics to have access to care plan, non-conveyance, one to three hours, epilepsy specialists today, GP notification, and specialist follow-up within two to three weeks. Family and friends differed when considering atypical seizures, favoring conveyance to urgent treatment centers and shorter time. Optimal configuration of services from service users’ perspectives outranked current practice. Knowledge exchange (n=27 participants) identified the optimal CP as feasible but identified two scenarios for resource reallocation: care plan substitutes specialist advice today and times of strain on NHS resources.

**Conclusions:**

Preferences differed to current practice but had minimal variation by seizure type or stakeholder. This study clearly identified optimal and feasible alternative CPs. The mixed-methods approach allowed for robust measurement of preferences, whilst knowledge exchange examined feasibility to enhance implementation of optimal alternative CPs in the future.

